# Global Causes of Diarrheal Disease Mortality in Children <5 Years of Age: A Systematic Review

**DOI:** 10.1371/journal.pone.0072788

**Published:** 2013-09-04

**Authors:** Claudio F. Lanata, Christa L. Fischer-Walker, Ana C. Olascoaga, Carla X. Torres, Martin J. Aryee, Robert E. Black, for the Child Health Epidemiology Reference Group of the World Health Organization and UNICEF

**Affiliations:** 1 Instituto de Investigacion Nutricional, Lima, Peru; 2 US Navy Medical Research Unit 6, Callao, Peru; 3 School of Medicine, Universidad Peruana de Ciencias Aplicadas, Lima, Peru; 4 Department of International Health, Johns Hopkins Bloomberg School of Public Health, Baltimore, Maryland, United States of America; 5 Division of Biostatistics, Sidney Kimmel Comprehensive Cancer Center, Johns Hopkins University, Baltimore, Maryland, United States of America; Tulane University, United States of America

## Abstract

Estimation of pathogen-specific causes of child diarrhea deaths is needed to guide vaccine development and other prevention strategies. We did a systematic review of articles published between 1990 and 2011 reporting at least one of 13 pathogens in children <5 years of age hospitalized with diarrhea. We included 2011 rotavirus data from the Rotavirus Surveillance Network coordinated by WHO. We excluded studies conducted during diarrhea outbreaks that did not discriminate between inpatient and outpatient cases, reporting nosocomial infections, those conducted in special populations, not done with adequate methods, and rotavirus studies in countries where the rotavirus vaccine was used. Age-adjusted median proportions for each pathogen were calculated and applied to 712 000 deaths due to diarrhea in children under 5 years for 2011, assuming that those observed among children hospitalized for diarrhea represent those causing child diarrhea deaths. 163 articles and WHO studies done in 31 countries were selected representing 286 inpatient studies. Studies seeking only one pathogen found higher proportions for some pathogens than studies seeking multiple pathogens (e.g. 39% rotavirus in 180 single-pathogen studies vs. 20% in 24 studies with 5–13 pathogens, p<0·0001). The percentage of episodes for which no pathogen could be identified was estimated to be 34%; the total of all age-adjusted percentages for pathogens and no-pathogen cases was 138%. Adjusting all proportions, including unknowns, to add to 100%, we estimated that rotavirus caused 197 000 [Uncertainty range (UR) 110 000–295 000], enteropathogenic *E. coli* 79 000 (UR 31 000–146 000), calicivirus 71 000 (UR 39 000–113 000), and enterotoxigenic *E. coli* 42 000 (UR 20 000–76 000) deaths. Rotavirus, calicivirus, enteropathogenic and enterotoxigenic *E. coli* cause more than half of all diarrheal deaths in children <5 years in the world.

## Introduction

Despite global success in the reduction of all cause and diarrhea-specific mortality in the past 30 years, diarrhea remains the second leading cause of death due to infections among children under five years of age worldwide [1], [2]. It is estimated that diarrhea accounted for 9·9% of the 6·9 million deaths among children under 5 in 2011 [2], [3]. Several organisms have been implicated as important causes of these deaths [4], [5], yet there has not been a review using standardized methods to determine the importance of all of the common pathogens. The Child Health Epidemiology Reference Group (CHERG) has estimated the causes of child deaths from major causes since 2001. We have undertaken this review to develop estimates of pathogen-specific diarrhea mortality among children under 5 years of age. We present the results of a systematic literature review of studies of diarrhea etiology in hospitalized children and use these results to estimate the global burden of diarrhea mortality by pathogen for children under 5 years of age for 2011.

## Methods

### Search strategy and selection criteria

We searched Medline, Lilacs, and MedScape for studies published between 1990 and 2011. We used the terms “diarrhea” (or “diarrhoea”), “gastroenteritis”, “rotavirus”, “E.coli” (or “Escherichia coli”), “Salmonella” (not “typhi”), “Shigella”, “Campylobacter”, “Giardia lamblia”, “Vibrio”, “Cryptosporidium”, “Entamoeba”, “norovirus”, “calicivirus”, “Norwalk agent”, using “AND children” as a search restriction. An example of one of the search instructions in Medline-PubMed is: “diarrhea”[mesh] OR “diarrhea”[all fields] or “diarrhoea”[all fields] OR “gastroenteritis”[mesh] OR “gastroenteritis”[all fields] OR “rotavirus”[mesh] OR “rotavirus”[all fields] OR “E.coli”[all fields] or “Escherichia coli”[mesh] OR “Escherichia coli”[all fields] OR “Salmonella”[mesh] OR “Salmonella”[all fields] OR “Shigella”[mesh] OR “Shigella”[all fields] OR “Campylobacter”[mesh] OR “Campylobacter”[all fields] OR “Giardia lamblia”[mesh] OR “Giardia lamblia”[all fields] OR “Vibrio”[mesh] OR “Vibrio”[all fields] OR “Cryptosporidium”[mesh] OR “Cryptosporidium”[all fields] OR “Entamoeba”[mesh] OR “Entamoeba”[all fields] OR “norovirus”[mesh] OR “norovirus”[all fields] OR “calicivirus”[all fields] OR “Norwalk agent”[all fields] AND “children”[all fields]. Limited to publication dates January 1, 1990 – December 31, 2011. We also included data from the WHO Rotavirus Surveillance Network for 2011 provided to us by WHO only from countries that had not introduced rotavirus vaccine as of December 2011 and had data covering the 12-month period. These studies used a standard protocol across the network [6]. We included studies that sought at least one of the above listed pathogens and conducted 12 or more months of surveillance among children less than 5 years of age hospitalized with diarrhea. Studies must have included all diarrhea patients at the selected study site or a systematic sampling of cases for the duration of the study. We did not require a minimal number of children evaluated to be included. Laboratory tests were performed on rectal swabs or stools samples. We excluded studies conducted during reported diarrhea outbreaks, those that did not discriminate between inpatient and outpatient cases, those that included patients with nosocomial infections, and those conduced in special populations, such as HIV-positive patients. We also excluded studies that did not describe adequate surveillance methods or standard laboratory methods, according to the following criteria: a) salmonella and shigella isolation in salmonella/shigella agar, xylose-lysine-deoxycholate agar, Hektoen enteric agar, and selenite enrichment for salmonella [7]; b) campylobacter isolation by use of transport media with antibiotics (Skirrow's supplement or similar) and inoculation into 5% sheep blood with antibiotics (Butzleŕs supplement or similar), cultivated at 42°C in micro-aerobic atmosphere [7]; c) *Vibrio cholerae* isolation by alkaline peptone water enrichment and subculture at 8 hrs into thiosulfate-citrate- bile salts – sucrose agar (TCBS) [7]; d) *E. coli* isolation from MacConkey agar and identification of ETEC by DNA probes or polymerase chain reaction (PCR) for heat-labile (LT) or heat-stable (ST) toxins, cell cultures (Y1, CHO cells), ileal loop or mouse models [7]; e) EPEC isolation by the use of Hep 2 cell cultures or the presence of the plasmid for adherence (BFP) and the intimin gene (eae) identify in DNA probes or by PCR [7]; f) rotavirus, calicivirus (or norovirus), astrovirus and enteric adenovirus identification with the use of enzyme-linked immunoassays (ELISA), electronic microscopy, or PCR [7]; g) *Giardia lamblia* identification by direct microscopic examination, or zinc-sulfate concentration from direct stools or by ELISA [7]; h) *Cryptosporidium* spp. identification by ELISA, or the modified Ziehl-Neelsen stain for microscopy [7]; i) *Entamoeba histolytica* identification by direct microscopic examination [7]. We did not include studies in areas or countries where the rotavirus vaccine was used but included data from the placebo arm of rotavirus vaccine trials. Articles published in languages other than English, Spanish, Portuguese, Italian, German and French were not included.

The following enteropathogens were considered: Rotavirus, enteropathogenic *Escherichia coli* (EPEC), enterotoxigenic *Escherichia coli* (ETEC), *Salmonella spp*. (excluding *Salmonella typhi*), *Shigella spp., Campylobacter spp., Vibrio cholerae* O1 and O139, *Giardia lamblia, Cryptosporidium spp., Entamoeba hystolitica*, human Caliciviruses (genogroup I and II norovirus and sapovirus) or astrovirus, coronavirus, and enteric adenovirus. We extracted data for all children less than five years of age for each pathogen. Data from more than one hospital in a country were treated as separate studies if the presentation of data permitted. Papers that published different etiological data from the same study site were grouped into one study. If co-infections were reported, they were not treated separately so each pathogen was counted as present if isolated alone or in combination. Three reviewers (CO, CXT, and CFL) did the primary extraction and all selected papers were reviewed by CFL and CFW independently. Disagreements were resolved by CFL and/or REB.

### Statistical analysis

We calculated overall median proportions of positive diarrheal stool samples for each pathogen for children 0–59 months of age using the overall proportion for all children included in the study; 39 studies enrolled children from a narrower age range so we calculated for these studies an age-adjusted proportion for the 0–59 months of age group by calculating a conversion factor for age group X as the median of 0–59 prevalence over age group X prevalence (median (prev_0–59_/prev_X_)) using studies that reported both 0–59 and the age group X for a given pathogen. To use this method we required at least 3 studies, where each study reported both 0–59 months and age group X. In situations where less than 3 studies were available we employed an alternative method where the conversion factor for age group X was taken as the ratio of the median prev_0–59_ to median prev_X_ (median (prev_0–59_)/median (prev_X_)). For this approach we required that 3 or more studies contribute to each of the two medians, but dropped the Method 1 requirement that individual studies report both age groups. If neither of these sets of conditions were met, we borrowed the conversion factor for the age group X from a similar age group within the same pathogen (for instance, used the conversion factor calculated for studies including infants 0–11 months of age for studies that included infants 0–5 months of age) or from a similar pathogen (conversion factor for age group X for a study on EPEC borrowed from studies on ETEC). The 0–59 months prevalence proportion for each pathogen was estimated using the median individual study 0–59 months pathogen prevalence.

We stratified studies by the number of pathogens sought and calculated the unadjusted and age-adjusted medians, as described above, separately for single pathogen studies and for studies that sought 5 to 13 pathogens. For estimating the proportion of diarrheal stools due to unknown pathogens, we included 12 studies that sought 8 or more pathogens.

For the numbers of diarrheal deaths attributable to each pathogen, we assume that the distribution of pathogens observed among children hospitalized for diarrhea represents the pathogen prevalence among child diarrhea deaths. We applied the age-adjusted median proportion for each pathogen and for unknowns to the overall number of diarrhea deaths of 712 000 estimated for the world in 2011 [3], adjusting all proportions equally to be constrained to add to 100%. We explored alternative estimates using all studies selected or only those that sought 5 to 13 pathogens, constraining or not all proportions to add to 100%. The uncertainty around each estimate was calculated using Bootstrap confidence intervals [8]. ‘Pseudo-data sets’ were created by sampling studies with replacement from the real dataset. Each of the 1000 pseudo-datasets was used in the estimation procedure described above to generate a corresponding 1 000 prevalence proportions. The 2·5^th^ and 97·5^th^ percentile of these proportions gave the 95% confidence interval (CI). To estimate the uncertainty of the number of deaths for each pathogen, we paired each of the 1 000 pseudo-datasets with random draws from the under 5 total mortality envelope, the proportion of total deaths attributable to diarrhea [2], [3], and the proportion of diarrhea deaths due to unknown pathogens. The under 5 year global total mortality envelope estimate and standard deviation were calculated by sampling and combining 100 000 random draws from each of the 194 countries in the world [2], [9]. For each country, a normal mean and standard deviation was estimated from the point estimate and associated confidence interval.

## Results

**Figure 1:**
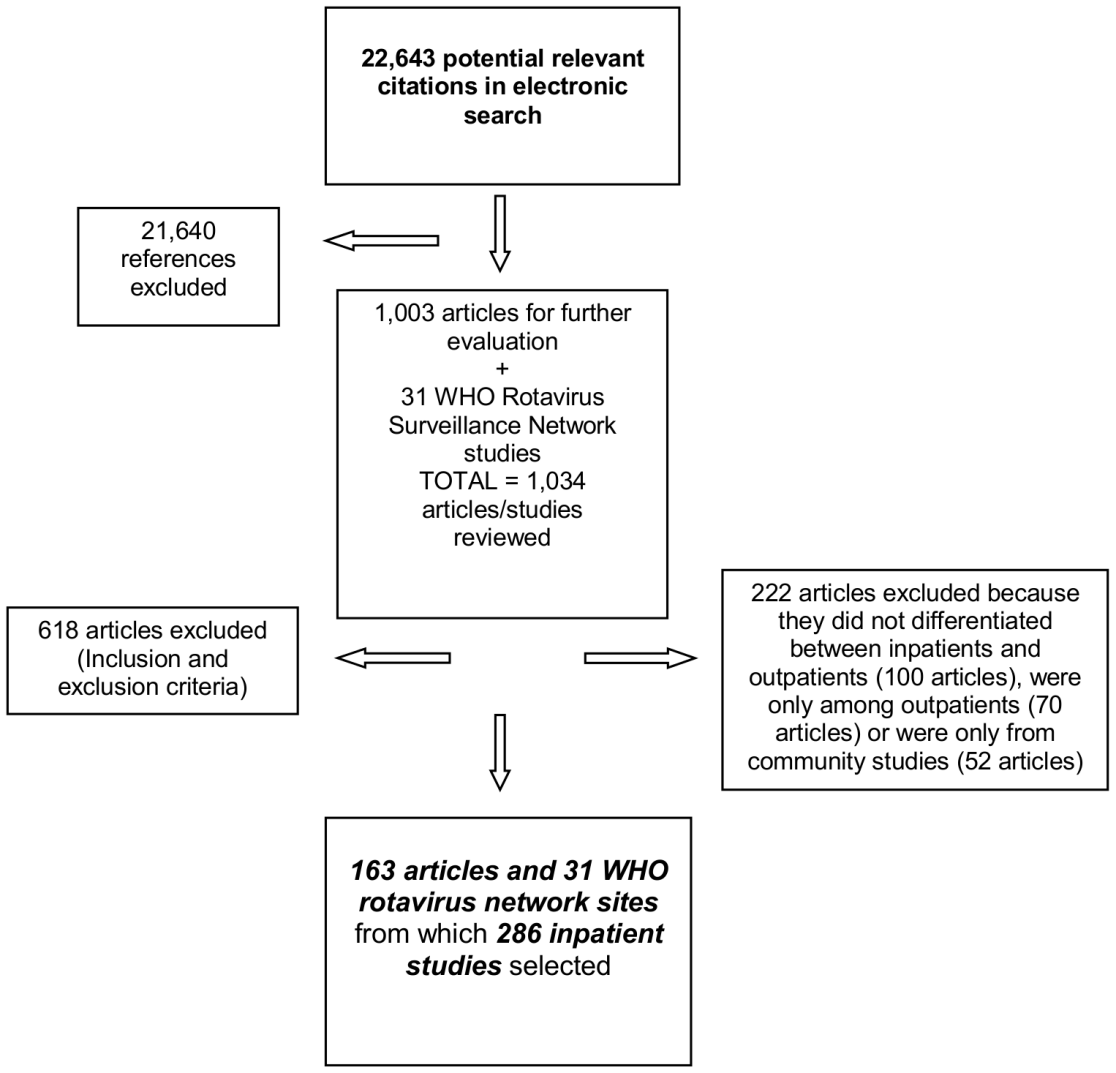
PRISMA Flow Diagram of studies included.

**Figure 2:**
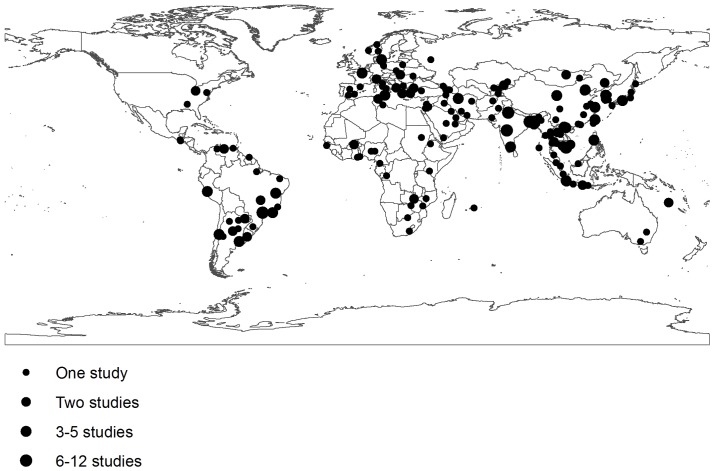
Location of the 286 inpatient etiologic studies included in the analysis.

From 22 643 citations identified in the electronic search, 1 003 articles were selected for further evaluation (Fig. 1); 840 articles were excluded because they had one or more of the exclusion criteria (About 35% because they were not longitudinal studies or inappropriate laboratory methods were used, 31% because no data was given for children <5 years of age, 23% for studies that lasted less than 12 months of duration, and the rest because data were reported after rotavirus vaccine introduction, duplicate publications or reporting results on a pathogen not included in our list). A total 163 articles and 31 WHO Rotavirus Surveillance Network sites were selected representing 286 inpatient studies with data for at least one pathogen [list of the 163 references can be found at www.cherg.org]. The geographical localization of the study sites is shown in Figure 2.

The median and age-adjusted median proportions (with 95% CI) of isolation of each enteropathogen in hospitalized diarrhea cases are shown in Table 1. Rotavirus, EPEC, calicivirus, and ETEC were the most frequently identified organisms. The sum of these age-adjusted median proportions, including unknowns was 138%, indicating a problem with many articles reporting mixed infections as separate causes. Different isolation rates were observed in studies in which only one, versus at least 5 enteropathogens were sought (Table 2). Rotavirus was more frequently isolated in 180 single-pathogen inpatient studies in comparison with 24 multiple-pathogen studies (39% vs. 20%, respectively, p<0·0001). The same trend was observed between single- and multiple-pathogen studies for most pathogens, but mainly for *Giardia lamblia* (16% vs. 3%, p<0·001), shigella (24% vs. 7%, p<0·001) and *V. cholerae* (10% vs. 0.2%, p<0·001). Very few studies sought a substantial number of pathogens. From the 286 inpatient studies, only 12 (4%) sought 8 or more pathogens (1 study with 13, 2 studies with 10, 5 studies with 9, and 4 studies with 8 pathogens). In these studies, 33·7% of cases had no pathogen identified.

**Table 1:** Age-adjusted median proportions of diarrheal episodes requiring hospitalizations associated with each enteropathogen in children 0–59 m of age in the world.

Pathogen	Inpatients (n = 286 studies)				
	N studies	N samples positive	N samples examined	Median %	Age adjusted median % (95%CI)
**Viruses**					
Rotavirus	242	77 392	228 277	38·2%	38·3% (35·5–40·2)
Calicivirus	36	4 468	52 179	13·6%	13·8 % (11·8–17·6)
Astrovirus	26	883	49 993	2·9%	3·0% (2·0–4·2)
Adenovirus	30	1 675	52 734	4·7%	4·3% (3·1–5·8)
**Bacteria**					
EPEC	11	708	4 461	15·3%	15·3% (7·8–27·6)
ETEC	21	1 032	18 989	6·9%	8·2% (4·8–12·2)
*Shigella spp*	36	946	66 502	4·7%	5·4% (2·9–7·9)
*Campylobacter spp*	32	951	54 580	4·3%	4·3% (3·0–8·8)
*Salmonella spp*	34	2 184	69 340	3·5%	3·5% (2·9–5·2)
*Vibrio cholerae* O1	19	1 024	51 043	1·8%	1·8% (0·0–6·1)
**Parasites**					
*Cryptosporidum spp*	25	517	46 254	2·7%	2·7% (0·6–5·6)
*Giardia lamblia*	17	536	40 444	3·1%	3·1% (0·0–14·2)
*Entamoeba histolytica*	15	175	55 365	0·3%	0·3% (0·0–3·5)

**Table 2:** Median proportions of pathogens isolated in stool samples from diarrheal episodes seen in inpatient services, in 208 studies that sought one or 27 studies that sought 5–13 pathogens in children 0–59 m of age in the world, by type of pathogen.

Pathogen	Single pathogen (n = 208 studies)					Studies that sought 5–13 pathogens (n = 27 studies)				
	N studies	N samples positive	N samples examined	Median %	Age adjusted median % (95%CI)*	N studies	N samples positive	N samples examined	Median %	Age adjusted median % (95%CI)
**Viruses**										
Rotavirus	180	59 226	161 126	39·4%	39·4% (37·1–43·1)	24	8 384	43 719	19·7%	20·2% (15·7–26·3)
Calicivirus	12	639	4 412	15·6%	15·6% (10·5–21·2)	7	2 681	39 195	8·2%	8·2% (4·8–12·7)
Astrovirus	1	28	708	4·0%	4·0% (NA)	10	577	39 597	2·3%	2.3% (1·1–3·5)
Adenovirus	1	17	866	2·0%	2·0% (NA)	10	942	39 615	3·6%	3·6% (1·7–5·8)
**Bacteria**										
EPEC	0	-	-	-	-	9	605	2 961	15·8%	15·8% (7·9–29·2)
ETEC	1	43	314	13·7%	13·7% (NA)	16	355	5 461	8·1%	8·2% (5·1–11·9)
*Shigella spp*	2	118	668	17·1%	24·5% (NA)	24	520	43 947	6·0%	7·2% (3·2–7·9)
*Campylobacter spp*	1	64	2 163	3·0%	3·0% (NA)	23	596	43 882	4·8%	4·8% (3·1–9·3)
*Salmonella spp*	0	-	-	-	-	24	853	44 060	3·2%	3·2% (2·7–3·5)
*Vibrio cholerae* O1	2	134	1 441	10·5%	10·5% (NA)	11	227	36 025	0·2%	0·2% (0·0–6·1)
**Parasites**										
*Cryptosporidium spp*	7	192	5 451	2·8%	2·8% (2·0–6·1)	17	290	40 493	2·6%	2·6% (0·4–7·0)
*Giardia lamblia*	1	46	291	15·8%	15·8% (NA)	14	425	39 762	2·8%	2·8% (0·4–10·5)
*Entamoeba histolytica*	0	-	-	-	-	12	150	39 067	0·3%	0·3% (0·0–3·8)

* CI =  confidence interval.

Adjusting all proportions, including unknowns, to add to 100%, we estimated that rotavirus caused 197 000 (Uncertainty range UR 110 000–295 000), enteropathogenic *E. coli* 79 000 (UR 31 000–146 000), calicivirus 71 000 (UR 39 000–113 000), and enterotoxigenic *E. coli* 42 000 (UR 20 000–76 000) deaths. These four pathogens were associated with 55% of all diarrhea deaths (Table 3). These estimates varied substantially depending on the methods used. If the proportions were not made to add to 100%, rotavirus would be said to cause 272 000 deaths or if only studies that sought >4 pathogens were selected and the proportions were adjusted to 100% rotavirus would be said to cause 126 000 deaths (Table 4).

**Table 3:** Number of diarrheal deaths estimated for each pathogen in children 0–59 m of age in the world for the year 2011, using constrained median proportions to fit 100%.

Pathogen	Medians restricted to add 100%		
	Median	No. Deaths (×1000)	95% CI (×1000)
**Viruses**			
Rotavirus	27·8%	197	110–295
Calicivirus	9·9%	71	39–113
Astrovirus	2·1%	15	9–26
Adenovirus	3·1%	22	12–37
**Bacteria**			
EPEC	11·1%	79	31–146
ETEC	6·0%	42	20–76
*Shigella spp*	3·9%	28	12–53
*Campylobacter spp*	3·2%	22	11–50
*Salmonella spp*	2·5%	18	10–30
*Vibrio cholerae* O1	1·3%	9	0–37
**Parasites**			
*Cryptosporidum spp*	2·0%	14	3–31
*Giardia lamblia*	2·3%	16	0–66
*Entamoeba histolytica*	0·2%	1	0–19
Episodes with unknown etiology	24·5%	176	56–304
**Total**	100·0%	712	491–1 049

**Table 4:** Number of diarrheal deaths estimated for each pathogen in children 0–59 m of age in the world for the year 2011, estimated using un-constrained or constrained median proportions to fit 100% obtained from all 286 inpatient studies or from 27 studies that searched for 5 to 13 pathogens.

	Using all 286 studies			Using 27 studies that searched for 5 to 13 pathogens					
Pathogen	Un-restricted medians			Un-restricted medians			Medians restricted to 100% including unknowns		
	Median	No. Deaths (×1000)	95% CI (×1000)	Median	No. Deaths (×1000)	95% CI (×1000)	Median	No. Deaths (×1000)	95% CI (×1000)
**Viruses**									
Rotavirus	38·3%	272	163–374	20·2%	144	82–206	17·8%	126	70–200
Calicivirus	13·7%	98	57–153	8·2%	59	28–115	7·3%	52	22–95
Astrovirus	3·0%	21	11–35	2·3%	17	7–27	2·1%	15	6–25
Adenovirus	4·3%	31	16–49	3·6%	26	10–44	3·2%	23	8–39
**Bacteria**									
EPEC	15·3%	109	43–213	15·8%	112	57–242	14·0%	99	51–196
ETEC	8·2%	59	28–102	8·2%	59	29–102	7·3%	52	24–92
*Shigella spp*	5·4%	38	17–71	7·2%	51	20–74	6·4%	45	17–69
*Campylobacter spp*	4·3%	31	16–71	4·8%	34	19–80	4·2%	30	15–73
*Salmonella spp*	3·5%	25	15–40	3·2%	22	13–32	2·8%	20	10–31
*Vibrio cholerae* O1	1·8%	13	0–49	0·2%	1	0–42	0·2%	1	0–36
**Parasites**									
Cryptosporidum spp	2·7%	19	4–45	2·6%	19	3–50	2·3%	16	2–43
*Giardia lamblia*	3·1%	22	0–97	2·8%	20	2–79	2·5%	18	2–66
*Entamoeba histolytica*	0·3%	2	0–29	0·3%	2	0–28	0·3%	2	0–24
Episodes with unknown etiology	33·7%	243	68–500	33·7%	243	68–500	29·8%	214	71–362
**Total**	137·6%	983	582–1 475	112·8%	808	491–1 244	100·0%	712	491–1 049

When classifying studies by WHO region, most studies were done in the Western Pacific Region (78 studies) and less in the Eastern Mediterranean Region (19 studies) (Table 5). Rotavirus was more frequently isolated in the Western Pacific Region (33%) and less in the American Region (23%). Other comparisons were limited by few or no studies in some regions (Table 5).

**Table 5:** Median age-adjusted proportions of causes of diarrhea, constrained to fit 100%, in 286 inpatients studies of children <5 years of age published between 1990–2011, by WHO region.

Pathogen	AFRO (n = 22)		AMRO (n = 53)		EMRO (n = 19)		EURO (n = 50)		SEARO (n = 64)		WPRO (n = 78)	
	N	Median	N	Median	N	Median	N	Median	N	Median	N	Median
**Viruses**												
Rotavirus	18	26·8	47	23·4	16	31·3	44	25·9	42	25·5	75	32·6
Calicivirus	1	15·9	6	13·6	1	10·2	11	9·8	6	8·4	11	10·3
Astrovirus	1	6·6	5	3·1	0	–	7	0·9	3	2·1	10	2·8
Adenovirus	1	3·7	4	2·4	0	–	10	2·7	4	5·1	11	3·4
**Bacteria**												
EPEC	1	10·3	6	10·8	2	13·0	0	–	2	8·9	0	–
ETEC	1	5·0	10	7·4	3	5·0	0	–	7	4·3	0	–
*Shigella spp*	2	4·1	15	5·7	2	11·9	5	0·1	10	3·5	2	0·2
*Campylobacter spp*	2	2·3	9	6·1	1	7·9	6	2·1	9	3·5	5	1·9
*Salmonella spp*	2	3·2	12	2·1	2	6·0	6	5·2	8	2·6	4	3·2
*Vibrio cholerae* O1	2	0·4	4	0·0	1	0·0	0	–	11	4·5	1	0·04
**Parasites**												
*Cryptosporidium spp*	2	2·5	12	3·1	0	–	1	0·0	9	2·1	1	0·3
*Giardia lamblia*	1	1·8	10	4·7	0	–	1	0·0	4	5·2	1	0·5
*Entamoeba histolytica*	1	0·3	8	0·02	0	–	1	0·0	4	1·7	1	0·2

## Discussion

In this review, we showed that more than half of the severe diarrhea episodes, most likely to result in death among children under the age of 5 years in 2011, could be attributed to rotavirus, EPEC, calicivirus, and ETEC. Our estimates have been adjusted for age in studies that did not cover all children <5 years old, and to add to 100%, including a fraction of episodes with unknown etiology. Such adjustments have not been done in previously published estimates for single diarrhea etiologies [4], [5], [10]–[12].

We identified a potential selection bias among studies that focus on a single pathogen. For example, the median proportion of diarrheal episodes with rotavirus identified varied from 39% in single-pathogen studies to 20% in studies that sought more than 4 pathogens. It is possible that studies looking for a particular pathogen are more likely to be conducted in a study site with a high prevalence of that pathogen and/or a low prevalence of other pathogens. An urban hospital that treats children of higher socioeconomic status and living in more hygienic conditions than children in rural areas may find a higher proportion of cases with rotavirus. A study of cholera done in a hospital in an endemic area may not be representative of national or regional populations. Because of the low number of studies that sought multiple pathogens, we have not restricted our analysis to only those studies, in an attempt to include as much global data as possible, but it should be recognized that the inclusion of single-etiology studies may result in a biased higher estimate for some pathogens.

By including 13 pathogens in this review we are able to address the problem of mixed infections, an important factor ignored in previously published single-pathogen estimates of deaths. No methodology has been developed to identify the true cause of an episode when more than one pathogen is identified in the stool. Our adjustment of all percentages to fit 100% is done to correct for this problem, assuming that each pathogen is equally likely to cause the illness. This is probably not correct because some organisms are carried in the feces for a relatively long time after infection-causing illness, like norovirus [13], or may not cause illness, especially in older children who have acquired immunity that protects against disease, but not carriage of the organism, like some protozoa [14]. This method of including all equally in the constraint to 100% of diarrhea deaths may result in an underestimate of the importance of some pathogens, such as rotavirus in young children, and overestimate the importance of others, such as Giardia. We do not have data on the presence of these pathogens in the stools of asymptomatic children in the studies selected in this review so we cannot determine the attributable fraction related to each pathogen as done in other studies [15]. However, controlling for pathogens found in non-ill children does not necessarily eliminate the problem because some pathogens with long excretion periods after illness, like norovirus, may be wrongly classified as not causing diarrhea. Carefully conducted longitudinal studies are needed to separate long-term excretors after illness from asymptomatic infections, to reveal the true pathogenic role of these different organisms in developing countries.

We estimated that the number of diarrhea episodes for which no pathogen can be identified is 34%, which is based on studies that sought at least 8 pathogens, not necessarily all 13 and thus may be an overestimate. These “unknowns” could be due either to the same pathogens not detected because insensitive methods were used to identify them (either the method itself or to using a rectal swab instead of a stool sample) [16], to the use of antibiotics prior to obtaining the stool sample, to other yet undiscovered infections, or to non-infectious causes of diarrhea. The proportion of samples with unknown causes was based on a selected group of 12 studies that searched for 8 or more pathogens. These studies do not represent the world as the rest of the studies did. The recently conducted studies called The Global Enterics Multicenter Study (GEMS) in 7 countries in Africa and Asia were designed to fill this gap [15], [17], [18]. However, they studied cases with moderate and severe diarrhea seen in health services (hospitals, emergency rooms and community clinics), not separating those being hospitalized from milder outpatient cases, therefore, those studies would not meet our inclusion criteria. Given that we cannot distinguish among the reasons no pathogen was found during the episode, our estimates may represent an under-estimate, at least for some causes. We could not include some pathogens known to cause diarrhea in our review, such as organisms that cause food-borne outbreaks (i.e. *Clostridium perfringens* [19], or *Staphylococcus aureus* producing enterotoxins [20]), because there are very little data on their importance in developing countries.

A recent review of rotavirus studies estimated that rotavirus caused 453 000 deaths in children <5 in 2008 [4]. If we would apply the median proportion of 38% rotavirus isolation found in the 242 inpatient studies that sought it in our review, without any adjustment, to the 1 236 million U5 diarrheal deaths in 2008, we would estimate 472 000 rotavirus deaths in 2008. In 2011 it is estimated that diarrhea deaths have been reduced to 712 000 [3]. Our estimate of 197 000 deaths due to rotavirus, using our improved methods, still represents an important global public health problem, with 23 children dying due to this condition every hour. This estimate does not account for any recent reduction in rotavirus-specific proportionate mortality due to the introduction of rotavirus vaccine, as seen in some Latin America countries [21], but these countries account for a very small fraction of global diarrhea mortality. Wide scale use of the rotavirus vaccine in high mortality countries will allow a more precise estimate of the true proportion of diarrhea deaths caused by rotavirus.

Our estimate of 28 000 deaths for shigella is much lower than a previous estimate of 667 695 deaths due to shigellosis in children under 5 years in the world in 1995 published by Kotloff et al [5]. This initial estimate was not based on a systematic review of the literature; rather, it used a single study in Latin America to estimate the proportion of shigella cases that were hospitalized and a Bangladeshi study to estimate the case-fatality rate of children hospitalized with shigellosis to estimate the global burden due to this organism. Using the same methodology of Kotloff et al but with an updated review of the literature and current case fatality rates observed in Bangladesh, Bardhan P et al [22] estimated that only 14 000 children younger than 5 years of age died due to shigellosis in Asia in 2005. Our estimates are compatible with this Asian estimate.

The total number of deaths due to calicivirus of 71 000 deaths has indicated to be the third most common cause of death due to diarrhea in children under 5 years of age. Few studies differentiated between GI and GII norovirus and other types of human caliciviruses, but in those few that did, most of calicivirus isolated in children with severe diarrhea have been due to norovirus GII [23], [24]. Patel et al [25] estimated 218 000 deaths due to norovirus among children under 5, but this was calculated using very different methods and assumptions: they used an attributable fraction due to norovirus when data on asymptomatic children was available, and applied their mean isolation rate of 12.1% from inpatient studies (not much different from our median isolation rate of 13.8%) to 1.8 million deaths due to diarrhea in the world; they did not adjust for mixed infections or unknowns.

The 79 000 deaths estimated to be caused by EPEC represent different sub-types of this type of pathogenic *E. coli*, a group that requires further epidemiological studies in different parts of the world to further characterize them since some sub-types are isolated with the same frequency in diarrhea and control children [26], new “typical” and “atypical” EPEC strains have been identified [27], and in some regions have been identified to cause more persistent than acute diarrhea [28].

These estimates have several limitations. The studies included in this review were conducted in selected sites and in some cases in populations with increased risk of diarrheal diseases. Thus, they may not be representative of the countries where they were conducted, nor of the world. For several regions, such as Russia and the former Soviet states or Sub-Saharan Africa we have limited or no data (fig. 2, Table 4). The gap of information from Africa, for pathogens other than rotavirus, is most acute because of the number of diarrhea deaths in this region is very high [1]–[3]. No study has been conducted to identify pathogens in children who died due to diarrheal diseases, so we assume that children in need of hospitalization are the best proxy of diarrhea deaths in low to middle income countries, but this may not be true for some pathogens. Another limitation is the combination of laboratory methods with different sensitivities to identify a pathogen: from the culture-based identification of salmonella or shigella to the highly sensitive real-time PCR method for norovirus. This may have affected the relative importance of one *vs* another pathogen in our estimates. We excluded studies on nosocomial infections, on displaced populations and on diarrhea outbreaks, which may have caused us to under-represent deaths due to some pathogens like *V. cholerae*.

**Figure 3:**
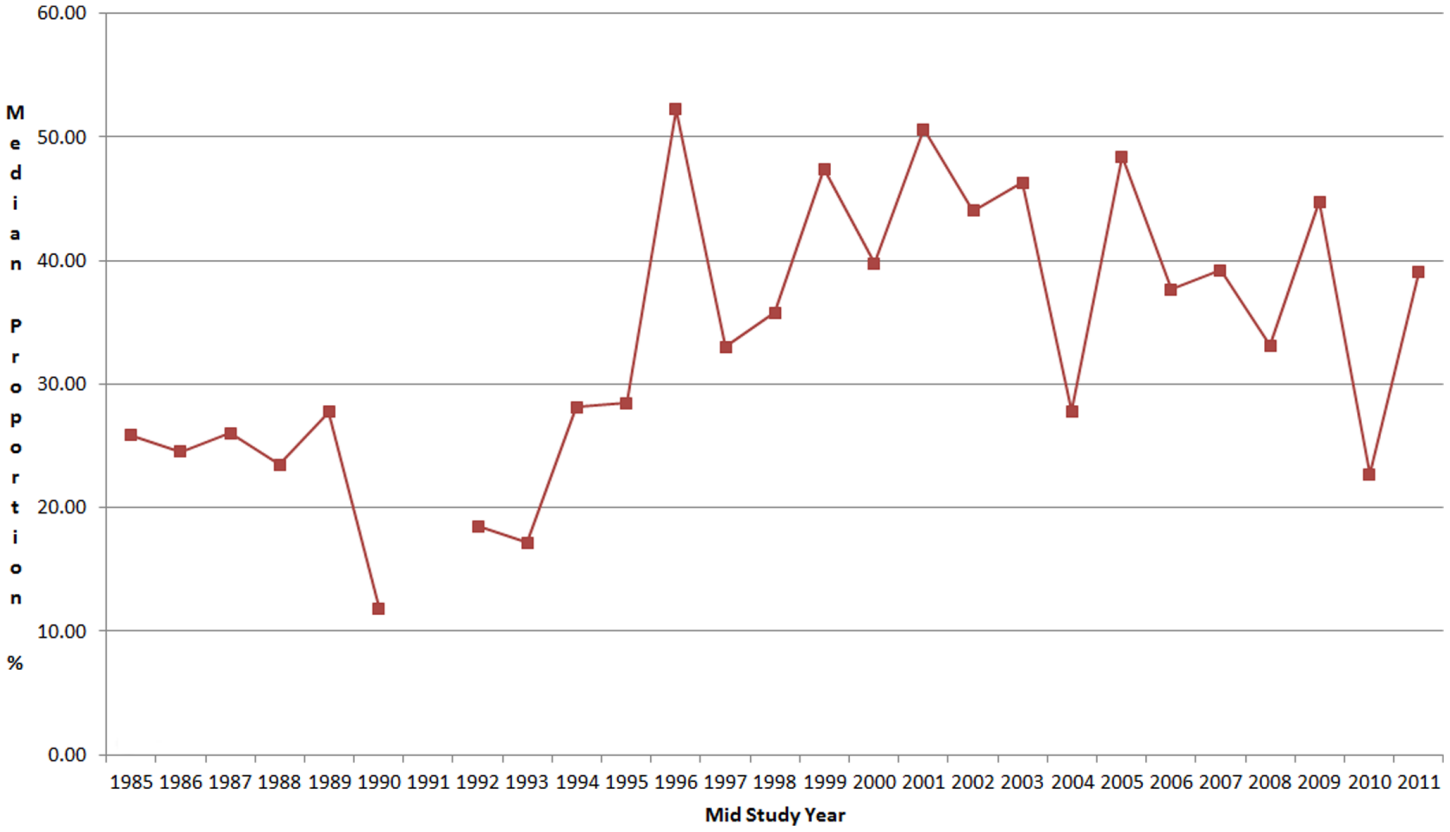
Median proportion of stool samples with rotavirus by mid study period.

We included in our estimates a total of 13 pathogens (4 viruses, 6 bacteria and 3 parasites) that have been incriminated as causes of severe diarrheal diseases. Some viruses, like adenovirus, and parasites, like *G. lamblia*, have not been completely documented as a cause of severe diarrhea in developing countries [14], [29], [30]. The subject of causality of diarrheal diseases is still not completely understood in settings where children are heavily exposed to many pathogens early in life. Young infants may be protected by breast milk and trans-placental maternal immunity and very low doses of ingested pathogens early in life may result in subclinical infections and development of immunity. This immunity may not preclude, however, the excretion of these pathogens in the child's feces. Practically all studies done in children who were studied when they were healthy as well as when they developed an acute diarrheal episode have found the same pathogens, although usually with lower frequency, in healthy states. Thus, the assumption that any pathogen identified in a child with diarrhea is the cause of the episode is naive and additional methods are needed to determine the pathogenicity of microbes. With a better understanding of the pathogenicity of key organisms our estimates could be further adjusted. Also, some studies suggest that children ill with a pathogen, as with EPEC, may excrete higher amounts in the stool, as compared with asymptomatic infections [31], so future studies may consider quantifying the amount of each pathogen in the stool to help identifying those ill with it. Finally, the review period covering studies published between 1990 and 2011 (studies were conducted with a median mid-study period of 2005, only 24 (8%) studies were done prior to 1990). We have not identified a significant change of the proportions assigned to each pathogen over time, so this does not seem to affect our estimates, as shown in Fig. 3 for rotavirus.

The Global Burden of Disease Study recently published cause of death estimates for 187 countries in 2010 [32]. For children <5 years of age, GBD estimated a total of 666 000 deaths due to diarrheal diseases in 2010 while CHERG estimated 712 000 deaths for 2011. GBD also estimated deaths due to 9 etiologies and produced estimates for 0–6, 7–27, and 28–364 days and 1–4 years of age. CHERG estimates for 2011 in children <5 years of age are slightly higher than GBD estimates for rotavirus (198 000 *vs* 173 000 rotavirus deaths, respectively), similar for EPEC and ETEC deaths (79 000 *vs* 73 000, and 43 000 *vs* 39 000, respectively), and lower for cholera, salmonella, shigella, campylobacter, *Entamoeba histolytica*, and *Cryptosporidium spp*. (Table 6). GBD did not estimate deaths due to norovirus, which was the third leading cause of death in our review. GBD used rates reported in diarrhea studies published between 1975 and 2010 done in outpatients, case-control, and community-based studies as a reference category to adjust the proportions seen in inpatient studies. CHERG only used data from inpatient studies published between 1990 and 2011. Both GBD and CHERG used modeling to obtain the total number of diarrheal deaths for children <5, but unlike GBD, CHERG has not used models for etiology-specific causes of deaths for each age group and for each country to produce its global estimate. Age specific data and modeling may produce spurious results, more so if there are no data. For example, very few studies have been done describing causes of diarrhea in neonates in developing countries, but GBD has estimated deaths caused by each of the 9 pathogens in neonates 0–6 and 7–27 days of age (Table 6). GBD only produced estimates for 9 etiologies of diarrhea and by subtracting the total of these estimates from the total of diarrheal deaths; they estimated the proportion of other causes of diarrheal deaths. CHERG estimated the proportion due to unknowns from studies that searched for 9–13 pathogens, which we feel realistically addresses the fact that a causative agent is not identified in every illness. This also explains why we estimated a higher number of deaths in this category (176 000) than GBD for “other causes” which should include unknowns (109 000). GBD and CHERG recognized the problem of mixed infections, but the methods used to adjust for it was different: GBD only used proportions for each etiology from inpatient studies that searched for 2–8 etiologies and used that information to produce weights to adjust their estimates in the models. We choose to constrain all proportions, including unknowns, to 100% to correct for mixed infections, which we feel it is more appropriate until better data and analytical tools are available. We have done an extensive search of the literature to include the 286 inpatient studies used in our estimates. GBD has not published the studies included, their search strategy, or modeling methods. Until these are published we will not be able to completely compare these estimates.

**Table 6:** Comparison between Global Burden of Diseases (for 2010) and the Child Epidemiology Reference Group (for 2011) estimates of the number of diarrhea deaths (×1000), by cause and age, in children <5 years of age in the world.

	Global Burden of Disease Estimate for 2010					CHERG estimate for 2011
Causes	0–6 d	7–27 d	28–364 d	1–4 yr	0–5 yr	
Total diarrheal deaths	25	52	353	235	666	712
*V. cholerae* O1	2	3	21	17	42	10
*Salmonella spp*	1	2	15	12	30	18
*Shigella spp*	2	3	22	17	44	28
EPEC	3	6	45	19	73	79
ETEC	1	3	20	14	39	43
*Campylobacter spp*	2	5	36	20	64	22
*Entamoeba histolytica*	0·3	1	4	4	9	2
*Cryptosporidium spp.*	4	7	47	25	83	14
Rotavirus	6	13	90	64	173	198
Other causes/unknown	4	8	54	44	109	176

This is the first systematic review attempting to estimate the cause of deaths for these 13 enteric pathogens. Rotavirus, calicivirus, enteropathogenic and enterotoxigenic *E. coli* cause more than half of all diarrheal deaths in children <5 in the world. We have identified a potential selection bias in studies searching for only one enteropathogen, and the problem when mixed infections (more than one enteropathogen is identified in a stool sample taken from a child with severe diarrhea) are not taken into consideration when estimating causes of diarrheal deaths, factors that has affected previous published estimates. Future studies should be done in hospital services dealing with all types of severe diarrhea, searching for all known enteropathogens, removing the effect of asymptomatic excretes, and establishing a mechanism to attribute to one enteropathogen the cause of a diarrheal episode in cases of mixed infections.

## Supporting Information

Checklist S1 ** PRISMA Checklist**
(DOC)

### Acknowledgments

Cynthia Boschi-Pinto of WHO and Theresa Diaz of UNICEF provided coordination of the involvement in CHERG of their respective institutions. Carolyn Weidemann served as coordinator of the grant in support of CHERG from the Bill and Melinda Gates Foundation. CHERG provided advice on methods and interpretation of results. We thank Walter Mendoza for his initial literature review, Cynthia Boschi-Pinto and Laura Lamberti for their support in searching for articles, and Edda Franco for editorial assistance. We thank the countries who provided data through the WHO-coordinated Global Rotavirus Surveillance Network of participating ministries of health, sentinel hospital sites, and the rotavirus laboratory network. We also thank John Sanders and Theresa J. Ochoa for providing useful comments on early drafts of the manuscript. Preliminary results of this study has been presented at CHERG and Food Borne Epidemiology Reference Group (FERG) meetings at WHO and at the Infectious Disease Society Association annual meeting in Vancouver, British Ontario, Canada on Oct 20^th^, 2010. **Disclaimer:** The views expressed in this article are those of the authors and do not necessarily reflect the official policy or position of the Department of the Navy, Department of Defense, nor the U.S. Government. Author Claudio F. Lanata is a contractor of the U.S. Government. This work was prepared as part of his official duties. Title 17 U.S.C. § 105 provides that ‘Copyright protection under this title is not available for any work of the United States Government’. Title 17 U.S.C. § 101 defines a U.S. Government work as a work prepared by a military service members or employees of the U.S. Government as part of those persons' official duties.
